# Contrasting effects of biochar and compost on greenhouse gas emissions and the global warming potential of semi-arid cropping systems

**DOI:** 10.1038/s41598-026-42554-4

**Published:** 2026-03-06

**Authors:** Piumi Madhuwanthi, Rajan Ghimire, Sundar Sapkota, Shannon Norris-Parish, April Ulery

**Affiliations:** 1 Department of Plant and Environmental Sciences, New Mexico State University, Las Cruces, NM USA; 2 Agricultural Science Center, New Mexico State University, 2346 State Road 288, Clovis, NM 88101 USA; 3 Department of Agricultural and Extension Education, New Mexico State University, Las Cruces, NM USA

**Keywords:** Soil amendments, Nitrous oxide emissions, Climate change, Dry environments, High-frequency monitoring, Biogeochemistry, Climate sciences, Ecology, Ecology, Environmental sciences

## Abstract

Agroecosystems in arid and semi-arid regions face growing risks of climate extremes and soil degradation. The addition of exogenous carbon can restore degraded soils by adding soil organic carbon, but its effects on greenhouse gas (GHG) emissions and global warming mitigation remain elusive. This study evaluated emissions of three major GHGs–nitrous oxide (N_2_O), carbon dioxide (CO_2_), and methane (CH_4_)–following soil amendment with biochar, compost, and a biochar + compost (BC) mixture. Biochar application reduced cumulative N_2_O–N and CH_4_–C emissions by 52% and 16%, respectively. Soil CH_4_–C emissions were generally negative, being lowest with biochar and highest with compost. During the crop season, average CO_2_–C and N_2_O–C emissions were 75% and 45% greater, respectively, while CH_4_–C was 66% less compared to the no-crop season. Increasing soil moisture content increased N_2_O–N emissions (*R*^*2*^ = 0.39), while soil temperature influenced CH_4_–C emissions (*R*^*2*^ = 0.37). Among amendments, biochar-treated soil had the lowest cumulative N_2_O–N and CH_4_–C emissions, reducing net global warming potential (GWP) by 43% and 30%, respectively, compared to compost-treated soil and control (CTRL). Biochar amendment can be a climate-smart strategy for semi-arid regions as it improves soil health and mitigates GWP by reducing N_2_O and CH_4_ emissions.

**Supplementary Information:**

The online version contains supplementary material available at 10.1038/s41598-026-42554-4.

## Introduction

The Earth is approaching a critical threshold of climate change, with projected global temperature increases of 1.5 °C by 2035 and 2.5 °C by 2100^1^. The steady increase in greenhouse gas (GHG) emissions poses a significant environmental concern, with notable impacts on the agriculture sector^2^. Paradoxically, the agriculture sector itself also contributes to GHG emissions and global warming. On a global scale, approximately 20% of direct GHG emissions arise from agriculture^3^, with soil management (e.g., tillage, fertilization, cover crops, amendments) accounting for the largest share^4^. The agriculture sector’s contribution was 11% of total GHG emissions in the USA in 2022^5^, with soil management alone resulted in approximately 0.3 Gt of carbon dioxide equivalent (CO_2_e) emissions^6,7^. The share of soil nitrous oxide (N_2_O) emission is the largest among GHG emissions (~ 75% of total ) from US agriculture. The N_2_O emissions occur during nitrification, denitrification, and other biological nitrogen transformation processes (i.e., co-denitrification, nitrate assimilation, dissimilatory nitrate reduction to ammonia, and chemo-denitrification)^8,9^. Soil CO_2_ is released during plant and microbial respiration, and CH_4_ is emitted mostly under anoxic conditions. As both a source and a sink of GHG emissions, precise monitoring and accurate quantification of soil fluxes is required to build an effective climate change mitigation plan^10^. However, data on soil GHG emissions are lacking for arid and semi-arid regions, which cover approximately 40% of the US landmass, predominantly in the western half of the country^11^.

Arid and semi-arid regions face numerous challenges in maintaining soil health and mitigating GHG emissions due to low biomass production, limited nutrient recycling, and high seasonal and interannual weather variability^12,13^. Extreme temperatures, prolonged drought, strong winds, and irregular precipitation—including hot-dry summers and cold-dry winters—can stimulate GHG emissions^14^. Exogenous carbon (C) addition through organic amendments, such as compost and biochar, may improve soil health and reduce global warming potential (GWP)^15^ by sequestering soil organic carbon. The amendments benefit soils by enhancing soil microbial activity, aggregate stability, water and nutrient retention, and infiltration capacity, mainly through elevated soil organic carbon content^16^. Specifically, the application of biochar, a C-rich material with a high surface area, can improve soil health, enhance water and nutrient retention, boost plant-microbe interactions, improve nutrient use efficiency, and ultimately support sustainable crop production^17^. However, their responses to GHG emissions are inconsistent; a study showed increased cumulative CO_2_–C emissions with dairy manure and reduced CO_2_–C and N_2_O–N cumulative emissions with biochar application under corn cultivation^18^. Another report showed soil CO_2_ emissions increased with higher application rates of organic amendments, irrespective of their type^7^. This inconsistency in soil GHG emissions stems from the variability in agroclimatic regions, cropping systems, management practices, crop inputs and outputs, and energy use during field operations^19–21^. Interestingly, GHG emissions following soil amendment applications are rarely reported from arid and semi-arid regions, where dry conditions typically result in low total emissions, whereas high weather fluctuations can lead to large GHG pulses after disturbance events. This highlights the need for region-specific GHG monitoring with the adoption of climate-smart soil management strategies. Alongside, estimating net GWP, which combines the radiative forcing effects of different GHGs over a defined period with the global warming factors of farm inputs and operations^22^, can help quantify the role of a given agricultural system in mitigating climate change^23^.

Soil GHG emissions have been measured using various methods, such as chamber methods, modeling, and micrometeorological methods^10^. Chamber techniques, including manual static chambers and portable smart chambers, have been widely used and are considered for the accurate estimation of fluxes. The static chamber technique requires manual gas collection and laboratory analysis (gas chromatography), while the portable chamber technique provides onsite flux measurements with minimal processing, but limited diurnal data. Moreover, chamber closure for a long period with manual sampling might disrupt concentration gradients, leading to biased flux measurements^14^. Both methods are labor-intensive, time-consuming, allow data collection mostly during daylight hours, and are not practical for quantifying short-term transient spikes of GHGs in response to rapidly changing environmental conditions and high temporal variations in gas emissions in semi-arid environments^14,24^. Additionally, the low or irregular frequency associated with these techniques often compromises annual emission estimates^25^. For instance, there are ample studies reporting net N_2_O sink by soils, but this process remains unclear due to the limited frequency of soil N_2_O emissions measurements. Additionally, soil moisture variation after periodic precipitation events and inaccessibility to fields make it challenging to estimate the flux variations, before, during, and after precipitation events^14^.

High-frequency GHG monitoring (several GHG flux measurements per day) has become possible due to the development of automated systems. This technique can identify major emission drivers throughout the year^14,26^ because these chambers enable reliable, continuous flux measurements that capture diurnal and seasonal emission variations in response to management and environmental changes. Additionally, this approach facilitates the measurement of both temporal and spatial flux dynamics with minimal soil disturbance. It enables measurement of real-time flux variations, flexible control of the chamber closure period, and data collection at user-friendly frequencies. However, the higher budgetary requirements and intensive maintenance have prevented the widespread use of these automated systems^27,28^. Nevertheless, we used an automated high-frequency GHG monitoring system to assess the GHG emission dynamics in amended semi-arid soils. This system allows long-term GHG data collection (CO_2_, N_2_O, and CH_4_), capturing diel variability, transient emission spikes, and seasonal dynamics driven by environmental changes (soil moisture, air and soil temperatures) and field operations (irrigation, fertilization, tillage, planting, harvesting, etc.). This study aimed to (1) evaluate GHG emissions after biochar and compost application in the depleted soils of the semi-arid environment of the southwestern USA to understand the diurnal and seasonal dynamics of GHG emissions, and (2) quantify the net GWP to assess the suitability of each amendment in mitigating climate change and sustaining soil health in water-limited environments. We hypothesized that GHG emissions and the net GWP would be reduced with biochar and compost amendments, based on previously reported work under similar environmental conditions and organic amendment applications.

## Results

### Soil greenhouse gas emissions dynamics

**Fig. 1:**
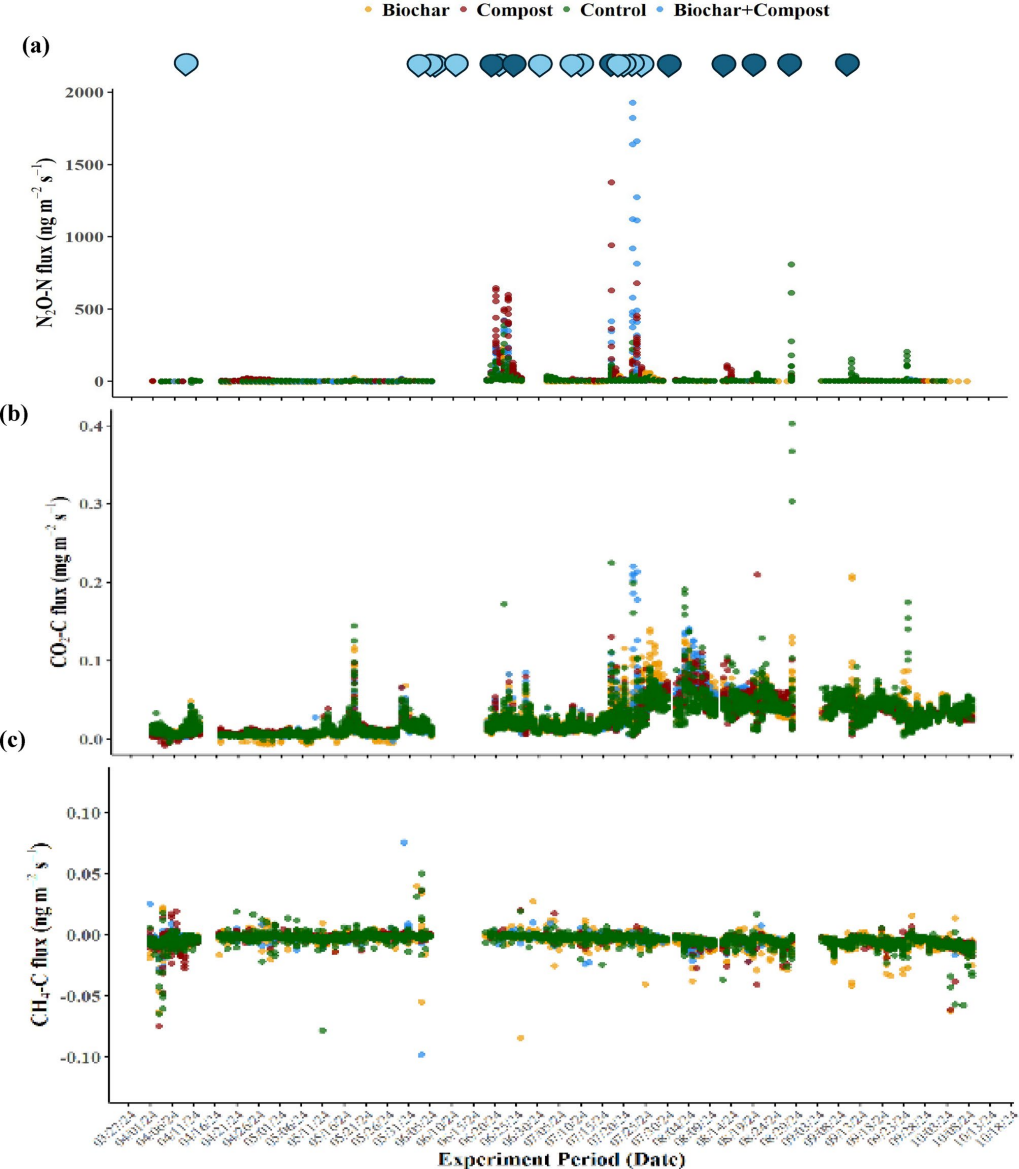
Variation in soil **a** N_2_O–N, **b** CO_2_–C, and **c** CH_4_–C emissions from April to October 2024. Dark blue drop marks represent irrigation events, and light blue drop marks represent rainfall events. Planting was done on 06/17/24, fertilization on 06/18/24, and harvesting on 10/16/24.

The trend in soil GHG emissions varied across treatments, with N_2_O–N and CO_2_–C showing clear responses to management and environmental conditions (Fig. 1). The emissions rates for CO_2_–C, CH_4_–C, and N_2_O–N ranged between − 0.01 and 0.37 mg m^− 2^ s^− 1^, – 0.10– 0.08 ng m^− 2^ s^− 1^, and − 12.6–1926 ng m^− 2^ s^− 1^ respectively, while median emissions for CO_2_–C, CH_4_–C, and N_2_O–N were 0.021 mg m^− 2^ s^− 1^, – 0.0026 ng m^− 2^ s^–1^, and 2.10 ng m^− 2^ s^− 1^, respectively (Table 1). Evaluating emissions trend for N_2_O–N showed peak emissions in July, primarily in compost (1,375 ng m^− 2^ s^− 1^) and BC (1,926 ng m^− 2^ s^− 1^) treatments, which were substantially higher than the 7-month average emission rates. In compost treatment, June and July average emissions were 77% and 17% greater than the 7-month treatment mean of 29.9 ng m^− 2^ s^− 1^, while in BC, those were 49% and 51% higher than the 7-month treatment mean of 33.1 ng m^− 2^ s^− 1^. Notably, 13% of N_2_O–N data showed negative emissions. Soil CO_2_–C emissions depicted minimal emissions from April to May (34% of total emissions), a gradual increase until August, and a declining trend thereafter (Fig. 1b). The CH_4_–C emissions were predominantly negative (94%), suggesting a net uptake across all the treatments (Fig. 1c). Among treatments, biochar resulted in the most negative emissions (– 0.0042 ng m^− 2^ s^− 1^) and BC the least negative (–0.0034 ng m^− 2^ s^− 1^). Soil water content and soil temperature also showed varying trends over the study months (Figure S1), with higher values during June–July and lower values during spring or fall seasons. The Soil water content was 0.10 m^3^ m^− 3^ and the temperature 2.83 °C greater than the 7-month average values (0.20 m^3^ m^− 3^ and 22.59 °C, respectively).

**Table 1:** Summary statistics for GHG emissions from April to October 2024.

Treatment	*n*	Mean	SD	SE	Median	Min	Max
*N*_*2*_*O–N* *Emissions (ng m*^*− 2*^ *s*^*− 1*^)							
Biochar	2,010	4.74	± 14.4	± 0.32	1.56	-9.53	230
Compost	969	29.9	± 100	± 3.24	3.19	-5.10	1,375
CTRL	1,494	10.4	± 37.5	± 0.97	2.63	-12.6	808
BC	813	33.1	± 160	± 5.62	2.11	-6.04	1,927
*CO*_*2*_*-C Emissions (mg m*^*− 2*^ *s*^*− 1*^*)*							
Biochar	3,497	0.03	± 0.03	± 4.70E-4	0.03	-0.01	0.91
Compost	1,822	0.03	± 0.03	± 6.40E-4	0.02	-0.01	0.81
CTRL	3,638	0.03	± 0.02	± 3.80E-4	0.02	-0.00	0.40
BC	1,348	0.03	± 0.03	± 7.60E-4	0.02	-0.00	0.22
*CH*_*4*_*-C Emissions (ng m*^*− 2*^ *s*^*− 1*^*)*							
Biochar	3,287	-4.05E-3	± 7.24E-3	± 1.00E-4	-0.003	-0.80	0.04
Compost	1,410	-3.65E-3	± 5.29E-3	± 1.00E-4	-0.003	-0.08	0.04
CTRL	3,293	-3.79E-3	± 4.90E-3	± 9.00E-5	-0.003	-0.08	0.05
BC	1,084	-2.22E-3	± 1.17E-2	± 4.00E-4	-0.002	-0.10	0.08

*CTRL = control, BC = biochar-compost mixture, SD = standard deviation, SE = standard error.

### Diurnal and seasonal variation in greenhouse gas emissions

**Fig. 2:**
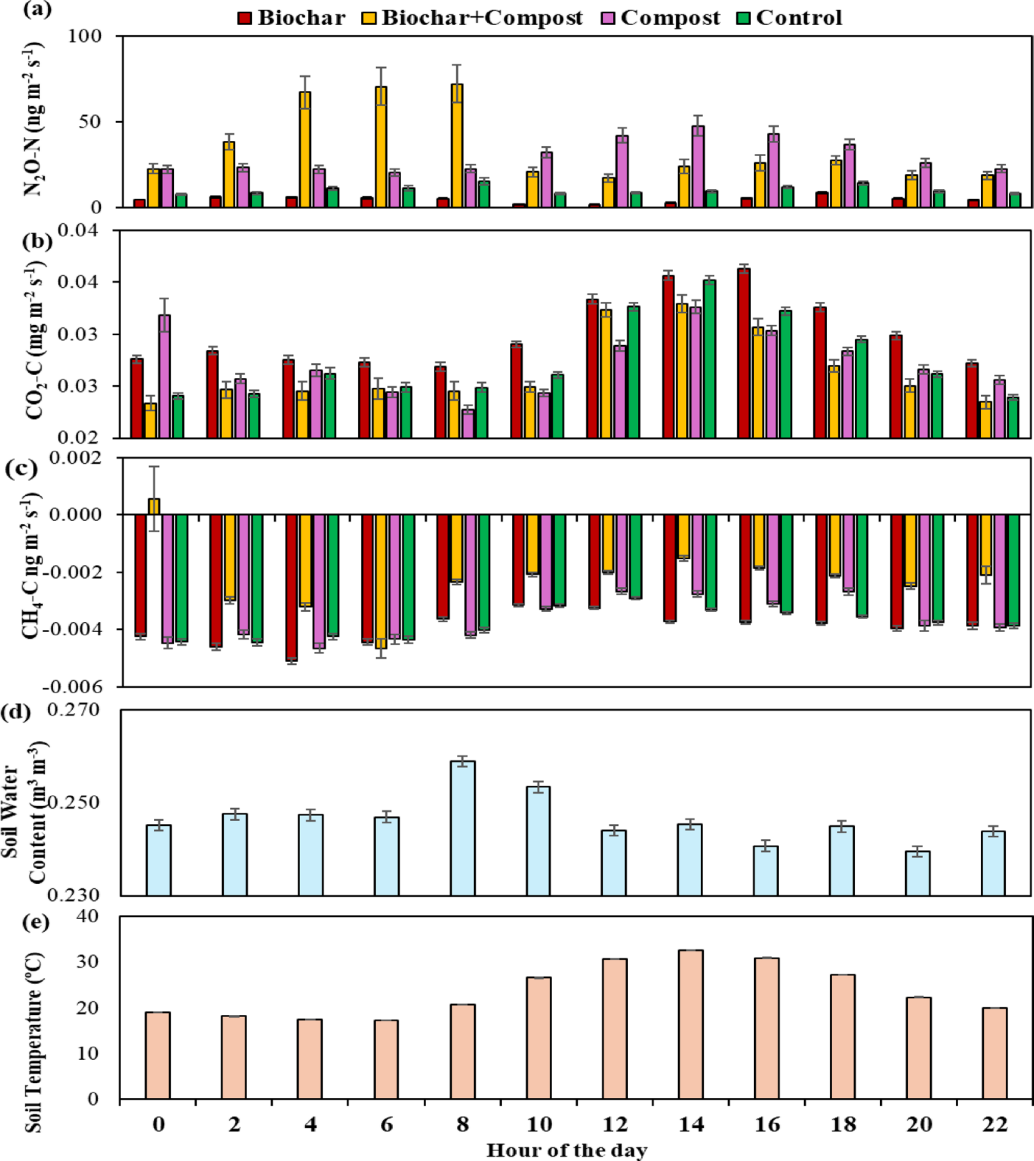
Average GHG fluxes for **a** N_2_O-N, **b** CO_2_–C, and **c** CH_4_–C, and variation in **d** soil water content, and **e** soil temperature within 24 h over the study period (April–October) in each soil treatment. Data are presented as mean ± standard error, and error bars indicate the standard error of each hour.

The diurnal trend of GHG emissions (average hourly emissions over the experimental period) showed a distinct trend in N_2_O–N emissions in compost and BC treatments. The BC showed peak emissions (Fig. 2a) during the morning (04:00–06:00 h) while compost had larger emissions during 14:00–18:00 h. Biochar and CTRL also followed the same pattern, but with much lower emission rates. The diurnal trend in CO_2_–C emissions was similar across treatments, with higher emissions during 12:00–18:00 h (Fig. 2b). Soil CH_4_–C also followed a diurnal trend, with less negative emissions during 10:00–12:00 h and more negative emissions during the morning and evening. The trend was similar across treatments, with biochar showing the largest negative emissions in most measurements (Fig. 2c).

**Fig. 3:**
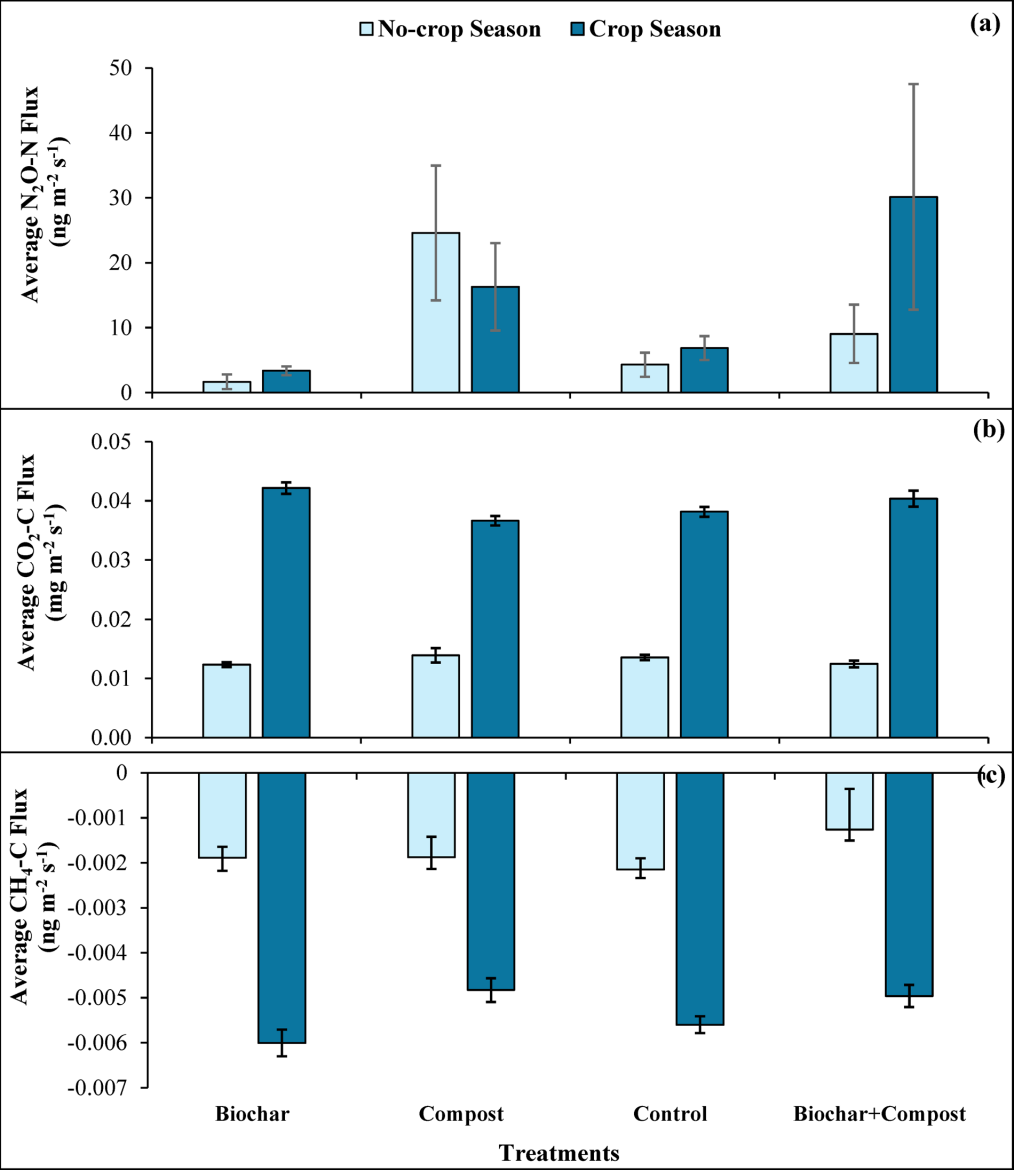
Average seasonal GHG emissions variation during the No-crop season (April–June) and Crop-growing season (July–October) for **a** N_2_O–N, **b** CO_2_–C, and **c** CH_4_–C, over the study period (April–October) in each soil treatment. Data are presented as mean ± standard error, and error bars indicate the standard error of each season for the respective treatment.

Seasonal variation in GHG emissions, examined by dividing the data into the no-crop season (April–June) and the crop season (July–October), showed highly variable emission rates in each period (Fig. 3). During the no-crop season, the N_2_O–N, CO_2_–C, and CH_4_–C average emissions ranged from 1.67 to 24.6 ng m^− 2^ s^− 1^, 0.011–0.013 mg m^− 2^ s^− 1^, and − 0.0013 to – 0.0022 ng m^− 2^ s^− 1^, respectively. No-crop season was characterized by relatively larger CH_4_-C emissions, while crop season represented more CO_2_–C and N_2_O–N emissions. During the crop season, the average emission of N_2_O–N, CO_2_–C, and CH_4_–C ranged from 3.37 to 30.1 ng m^− 2^ s^− 1^, 0.037–0.044 mg m^− 2^ s^− 1^, and − 0.0048 to – 0.0060 ng m^− 2^ s^− 1,^ respectively, which was 45% more N_2_O–N, 75% more CO_2_–C, and 66% less CH_4_–C compared to the no-crop season.

Soil temperature and moisture content played a significant role in regulating diurnal and seasonal changes in GHG emissions. For example, nonlinear regression analysis of soil water content with GHGs revealed positive, statistically significant relationships (Figure S2). Soil N_2_O–N and CO_2_–C emissions exhibited positive correlations with soil water content (*R*^2^ = 0.39 and 0.08, respectively, and *p* < 0.001). Soil CH_4_–C emissions also showed a significant relationship with soil water content, with slightly stronger *R*^2^ with untransformed data (*R*^2^ = 0.08, *p* < 0.001) than with log-transformed data (*R*^2^ = 0.03, *p* < 0.001). Most N_2_O–N emissions occurred in a volumetric soil water content range of 0.15–0.30 m^3^ m^− 3^, whereas CO_2_–C emissions spanned a broader range (0.05–0.35 m^3^ m^− 3^), and CH_4_–C were largely confined to 0.20–0.30 m^3^ m^− 3^. Similarly, soil temperature showed significant positive relationships with all three gases, with *R*^2^ values of 0.08, 0.23, and 0.37 for N_2_O–N, CO_2_–C, and CH_4_–C, respectively (*p* < 0.001). Peak N_2_O–N and CH_4_-C emissions were observed with a soil temperature range of 15–25 °C, while CO_2_–C emissions occurred over a broader range (10–30 °C).

### Cumulative greenhouse gas emissions and net global warming potential

Cumulative emissions of CO_2_–C, N_2_O–N, and CH_4_–C for the study period varied among treatments (Table 2). The biochar contributed the largest CO_2_e from CO_2_–C among treatments, which were 11% and 1% greater than compost and CTRL, respectively. The cumulative CH_4_–C emissions were negative across all treatments. In contrast, the highest CO_2_e from N_2_O was observed in compost, with biochar emitting 85% and 52% less N_2_O than compost and CTRL. Our calculations showed the indirect effect of N fertilizer addition alone was 0.45 kg CO_2_e ha^− 1^. The CH_4_-based CO_2_e was negative across all the treatments, with the biochar treatment being 16% lower than CTRL. The sum of CO_2_e was the highest in the biochar treatment, exceeding that of the CTRL by only 0.08%. However, crop production, inputs, and soil respiration also contributed to GWP. Notably, BC plots had the highest cumulative sequestration (past year crop residue + C inputs to soil), 17% greater than CTRL. Additionally, N fertilizer application represented a significant share of net GWP across all the treatments (5579 kg CO_2_e ha^− 1^ yr^− 1^) (Table S1). Therefore, net GHGI was greatest in compost, i.e., 18% greater than CTRL, while the biochar treatment had the lowest GWP and GHGI.

**Table 2:** Cumulative emissions of each greenhouse gas (April–October 2024), cumulative CO_2_e calculated at a 100-year scale, and net global warming potential for each soil treatment.

Treatment	Cumulative emissions (kg ha^− 1^)				CO_2_e (100-year scale, kg ha^− 1^)					
	CO_2_-C	*N*_2_O-*N*	CH_4_-C	Sum of all	CO_2_	*N*_2_O	CH_4_	Sum of all	Net GWP (Mg CO_2_e ha^− 1^ yr^− 1^)	GHGI (kg CO_2_e kg^− 1^ of dry grain yield)
Biochar	4,865	0.36	– 0.66e^− 3^	4,865	17,829	155	– 24.9e^− 3^	17,984	6.84	0.51
Compost	4,351	2.34	– 0.43e^− 3^	4,353	15,944	1004	– 16.2e^− 3^	16,948	11.9	0.97
CTRL	4,816	0.75	– 0.55e^− 3^	4,817	17,646	324	– 20.9e^− 3^	17,970	9.8	0.80
BC	4,546	2.09	– 0.45e^− 3^	4,548	16,660	898	– 17.1e^− 3^	17,558	7.26	0.53

†Carbon dioxide-equivalents (CO_2_e) obtained by the conversion factors of 273 for N_2_O, 28.5 for CH_4,_ and 1 for CO_2_ for a 100-year scale (IPCC, 2023a), GWP = global warming potential, GHGI = Greenhouse gas intensity.

## Discussion

### Management and environmental drivers of greenhouse gas emissions

Soil-plant-microbe interactions influence soil GHG emissions in agroecosystems. In this study, interactions among soil moisture, oxygen (O_2_) concentrations, temperature, plant roots, and C and N substrates regulated N_2_O emissions. Specifically, high-emission pulses observed in June/July - up to a 98% increase across treatments - indicate the microbial community’s response to the combined effects of soil water and temperature. Microbial transformations of reactive N (nitrification, denitrification, and dissimilatory NO_3_^−^ reduction) generate N_2_O from soil^30^, the relative emissions varying with soil characteristics and prevailing environmental conditions. Trost et al.^31^ reported a 50–140% increase in N_2_O emissions after summer rain, mainly due to soil degassing (water entering soil pores displacing soil air). In dryland systems, nitrification dominates as a source of N_2_O at low water content, which equilibrates with denitrification when soil moisture reaches the peak of the oxic-anoxic interface after irrigation or summer rain, and ultimately shifts to denitrification under higher soil water content^32^. Therefore, the high emission pulses after water input may have resulted from a combination of nitrification, denitrification, and physical degassing. We also noted some negative values (net N_2_O uptake), which can occur in any water-filled pore space between 25% and 80%, but mainly when it is relatively dry (approximately WFPS 5–20%)^33,34^. At this point, N_2_O reduction outpaces production, typically due to aerobic denitrification or nitrifier-denitrification^34^.

Soil temperature was the primary regulator of soil CO_2_–C emissions, while soil moisture and aeration further influenced the emission dynamics^35^. In general, increasing soil moisture content increased CO_2_ emissions^36,37^, but we observed only a weak relationship, probably due to limited water availability in this study. Moreover, the temporal dynamics of water availability—amount and timing of rainfall events – impacted soil respiration more than soil moisture content. An increase in soil moisture can reduce the O_2_ concentration in soil pores (reduce gas diffusivities), interfering with microbial respiration. However, biochar induced higher emissions of CO_2_, most likely attributed to higher soil temperature and increased air-water exchange in porous biochar amendment than other amendments, leading to higher microbial respiration^38–40^.

The CH_4_ emissions were affected by the same factors as CO_2,_ while CH_4_-C emissions were predominantly negative or near-zero (94% of the total), with increased CH_4_-C uptake during dry, warm periods. Soil moisture and CH_4_ emission in arid or semi-arid soils generally show a positive relationship^41,42^. Although rainfall and water application temporarily increase CH_4_ uptake in dryland soils, overall increases in soil moisture decrease CH_4_ uptake or increase emissions^43^. Reduced CH_4_ uptake at higher soil moisture is likely due to slower diffusion of CH_4_ and O_2_ in the soil, limiting activities of methanotrophs^44^. Additionally, more negative CH_4_-C emissions observed during morning and evening hours likely followed the diurnal soil temperature variations in our study. Elevated soil temperature during warmer months enhances methanotrophic activity and increases the rate of CH_4_ uptake^41^. Warming affects the activity of CH_4_-producing and oxidizing bacteria in soil by altering soil temperature, moisture, nutrient levels, and permeability^45^.

Soil amendment specific changes in the C: N ratio also altered GHG emissions by affecting substrate availability and enzyme activities. For instance, N_2_O emission spikes were observed immediately after fertilization, especially under higher NO_3_^−^ availability and lower C: N ratios in compost and BC treatments. Here, the relative abundance of labile C and N fractions can determine nitrification rates and nitrite (NO_2_^−^) accumulation, a precursor of N_2_O through nitrification and nitrifier denitrification^46^. A relatively low C: N ratio in compost can lead to incomplete denitrification, producing more N_2_O under favorable conditions^47^. In contrast, biochar reduced N_2_O emissions by sorbing soil NO_3_^−^ due to its higher surface area and organic matter sorption capacity^30^. A meta-analysis reported this reduction to be approximately 33% ^48^, while our results show a substantially greater reduction of 52%. However, compost addition along with biochar introduces significant amounts of readily available N, which may override the N_2_O-mitigation potential of biochar^49^. Biochar-induced higher CO_2_ emissions are likely due to the labile C input and positive priming effects^48^. Although previous work reported this increment to be approximately 22% ^48^, the current study observed smaller increments of 1% with biochar and 7% with BC, possibly due to low-fertility soil and semi-arid environment. The observed CH_4_-C uptake with biochar can be due to the enhanced adsorption of CH_4_ to biochar surfaces and increased diffusive uptake with enhanced aeration^50,51^. Additionally, the increased O_2_ supply reduced the activity of methanogenic microorganisms, resulting in lower CH_4_ emissions^48,52^. We observed greater negative CH_4_ fluxes in the morning and evening under biochar treatment, likely due to enhanced soil temperature and moisture effects from biochar applications. A similar trend has been reported by Yeboah et al. ^53^, with average uptake ranging from 17 to 119%, varying with the biochar application rates. However, previous work reported inconsistent CH_4_ uptake rates varying with soil moisture levels, soil types, and climatic conditions^54^. In contrast, the labile C pool fraction of biochar can serve as a substrate for methanogenic activity, promoting CH_4_ production, when moisture is not limited^50^. Low soil organic matter content reduces CH_4_ uptake by limiting aggregate formation and pore space for methanotrophs^41^.

### Plant-mediated changes in greenhouse gas emissions dynamics

**Fig. 4:**
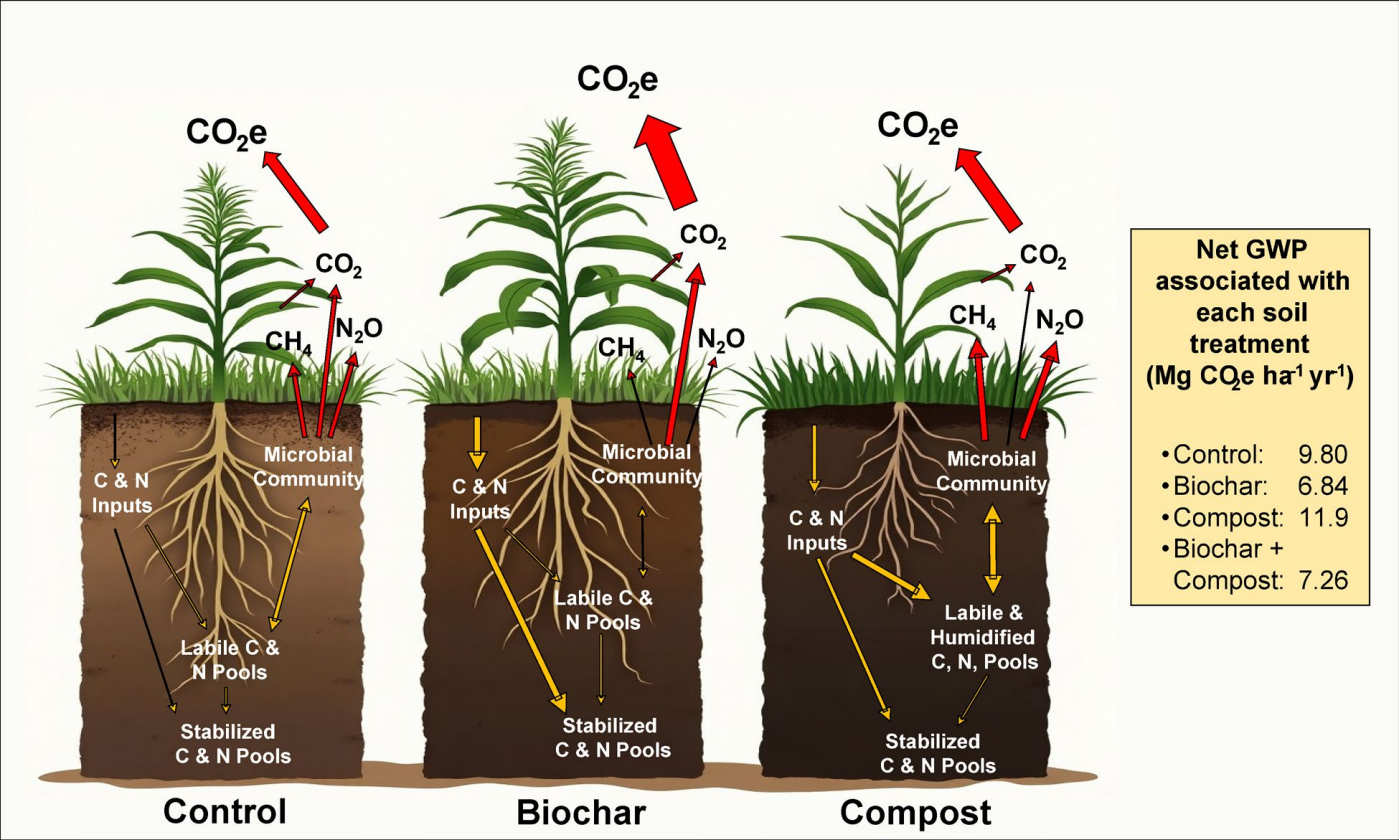
The illustration conceptually represents the soil C and N dynamics associated with each soil treatment relevant to soil GHG emissions. Red arrows denote the emission pathways of GHG, while yellow arrows indicate the processes such as mineralization and decomposition. The varying sizes of the arrows reflect the relative sizes of the corresponding nutrient pools or emission fluxes. Net GWP values are derived from the cumulative CO_2_e generated at each farm-related activity in each soil treatment. Image generated using Leonardo. Ai, based on text prompts provided by the author^64^.

Plant roots are a major contributor to soil respiration and, thereby, to overall GHG emissions from agroecosystems^55^. Our observations of a notable difference in GHG emissions between the crop and no-crop seasons, with only 22% of the total cumulative CO_2_e emissions from CO_2_–C occurring during the no-crop season (Table S2), indicate low microbial activity during the no-crop season. Rhizosphere respiration is null, and soil microbial activity is low during the no-crop season due to a lack of root exudates and limited root turnover^56^. Additionally, N_2_O–N emissions during the no-crop period were reasonably low (Fig. 3), possibly due to the low mineralization rate of residual N from the previous crop season and poor native N mineralization^57^. In contrast, soil abundant in N substrates enhances soil microbial activity by boosting nitrification and denitrification processes (Fig. 4). If labile N resulting from N transformation is not utilized by plants, it is prone to loss as N_2_O. Greater N availability in compost and BC plots led to greater N_2_O emissions during fallow. For CH_4_, we did not see much response from management, and emissions were negative during the no-crop season. Arid and semi-arid soils often act as a net CH_4_ sink, with magnitudes varying between the no-crop and crop seasons^58^.

During the crop (sorghum) season, emissions increased with the growth and development of the crop. For example, emission peaks in the July-August period were a result of the rapid turnover of above- and belowground biomass^55^. Rhizodeposition also increases as crops grow, contributing to microbial growth and increased heterotrophic respiration^59^. In addition, plant root respiration accounts for a significant portion of the total CO_2_ emissions (10–90%), varying with the plant’s growth stage, vegetation type, soil, and climatic conditions^55,59^. The CO_2_–C emissions in the current study (Fig. 4) are consistent with Nilahyane et al.^55^, who reported a 46–70% increase under crop growth compared to fallow. The elevated N_2_O–N emissions observed at the onset of crop season were likely due to rapid N mineralization following N fertilizer application. However, N uptake is also high as sorghum grows rapidly between the third growth stage (30–40 days post-emergence) through growth stage 6 (half bloom^60^;), reducing N loss as N_2_O emissions. Soil CH_4_-C emissions are mostly negative, as soil acts as a sink during the crop season^61^, but increased soil aeration under biochar further supported CH_4_ assimilation and reduced GWP. The negative emissions in arid and semi-arid soil are attributed to the prevailing drier aerobic conditions, limiting the activity of methanogens, and favoring CH_4_ oxidation^62,63^.

### Net global warming potential and policy implications

Net GWP and GHGI are determined by the balance between soil N_2_O and CH_4_ emissions, and C inputs^65^. Energy use and CO_2_ emissions associated with common agronomic activities were consistent across treatments in our study (Table S1). Therefore, the differences in total CO_2_e among the treatments were due to specific treatment applications. For example, biochar amendment resulted in the lowest net GWP, suggesting its greatest potential to mitigate climate change. Biochar production–from feedstock collection to final processing–typically leads to a higher net CO_2_ emission (~ 250 tons CO_2_e). However, their high C: N ratio, along with their ability to alter other soil properties, leads to reduced N_2_O and CH_4_ emissions and the lowest net GWP. In the long run, biochar applications can be environmentally sustainable^66^. However, the major drawback of biochar application is the higher cost associated with biochar production (from cradle to gate) and transportation, which limits its widespread use across regions. Additionally, variability in feedstock, pyrolysis conditions, and application rates, together with climate and soil texture, has been reported to yield inconsistent results. Compost applications, on the other hand, can increase net GWP, mostly due to CO_2_e generated during compost production and the rapid decomposition of C (especially during the summer period), more than biochar. Compost decomposition rate may vary depending on feedstock type, composition, and environmental conditions. Nevertheless, it can lose a significant amount of C as CO_2_ in a short time. Yun et al.^67^ reported that cattle manure compost loses about 72% of its C within 40 months. Compost application can also result in heterogeneity and variations in quality, as well as the potential introduction of pathogens and weed seeds. Unlike biochar, compost is more widely available and less expensive. However, it requires repeated applications to meet crop requirements due to rapid nutrient loss, potentially increasing long-term costs.

Given biochar’s potential to reduce soil N_2_O and CH_4_ emissions and enhance overall soil properties in the semi-arid soils, policies should incentivize its proper application. In the long run, biochar amendment may result in a sustained reduction in net GWP. Given the region’s exposure to prolonged strong winds, it is important to consider potential losses of applied biochar, underscoring the need for improved application strategies. Additionally, it is necessary to consider biochar aging and its long-term effects on soil GHG mitigation, given the greater discrepancy and limited published work. Although many studies have reported yield benefits with biochar application, additional research in semi-arid regions is essential to fully explore its potential. Moreover, research should examine the various effects of biochar prepared using different techniques and conditions to ensure optimal mitigation of GHG emissions and net GWP. Another concern is the slow nutrient release rates and potential N immobilization, which might limit adequate nutrient supply for crop growth. Likewise, understanding the drivers of GHG emissions in arid and semi-arid systems, along with identifying region-specific limitations, will help develop efficient models that yield robust predictions of GHG emissions across varying climatic conditions. It is also essential to investigate the complex interactions between different types and amounts of biochar applications and their long-term effects on the soil-plant-microbe interface. This study revealed that biochar is a promising soil amendment to enhance climate change mitigation in semi-arid soils.

## Conclusion

Greenhouse gas emissions in semi-arid cropping systems are primarily regulated by soil moisture, temperature, amendments, and plant-rhizosphere related interactions. Soil respiration responses to hydrothermal conditions governed soil CO_2_–C emissions, with wetting events stimulating soil microbial activities, particularly during dry periods. Soil amendment properties further modified these responses, with biochar reducing the emissions compared to compost. Soil N_2_O–N emissions closely followed soil moisture and temperature variations, peaking after precipitation or irrigation events due to induced nitrification and denitrification processes. Biochar application reduced these emissions, whereas compost enhanced emissions with increasing soil moisture, reflecting the higher lability of substrate. Soil CH_4_–C emissions were minor and often negative, indicating a net CH_4_ sink (uptake) in semi-arid agroecosystems, and were sensitive to soil hydrothermal dynamics. Overall, this study emphasized the importance of soil moisture regulation and the selection of organic amendments for mitigating GHG emissions in semi-arid agroecosystems. The use of high-frequency GHG monitoring revealed diurnal and short-term dynamics that are often overlooked, underscoring the importance of context-specific temporal monitoring for reliable emission estimates for sustainable climate-smart management strategies in semi-arid regions.

## Methods

### Study site and treatments

**Fig. 5:**
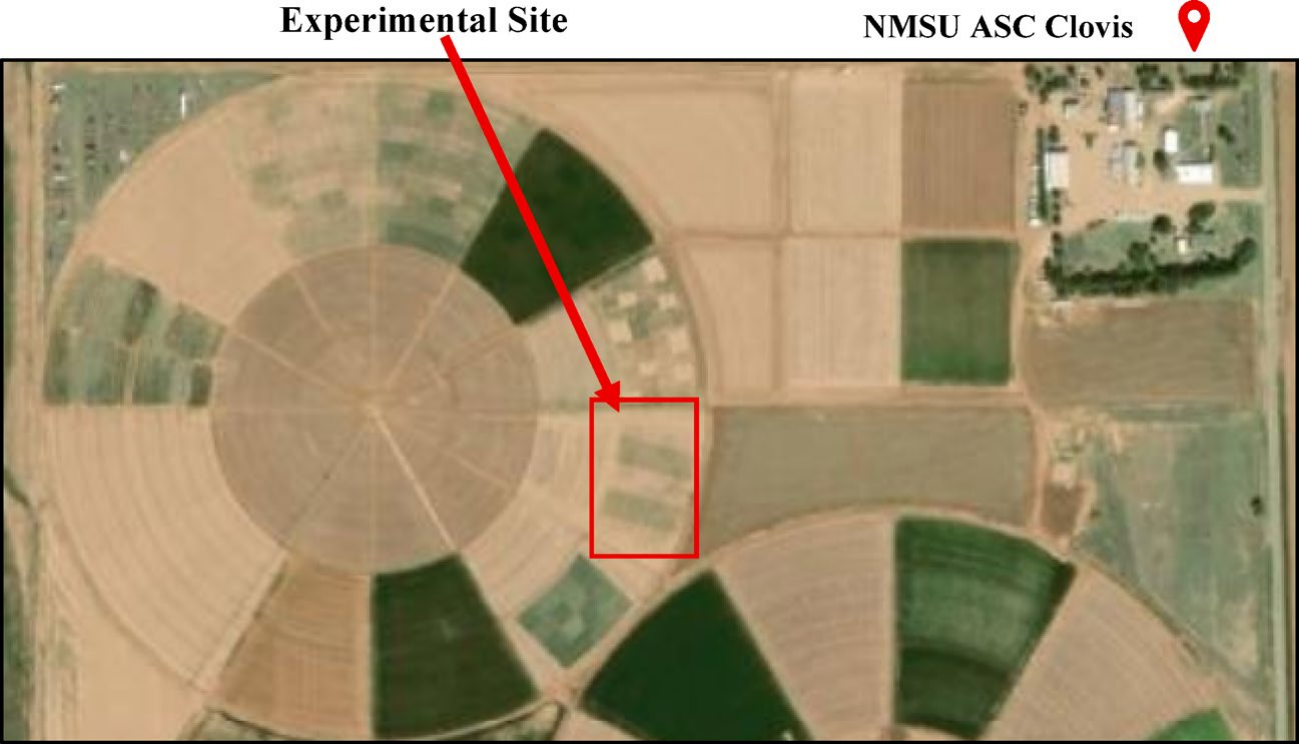
Experimental site at New Mexico State University Agricultural Science Center (NMSU ASC) at Clovis, NM. (Source: https://earth.google.com/)

A long-term field experiment was established in March 2024 at New Mexico State University Agricultural Science Center at Clovis, NM (34°35′ N, 103°12′ W, elevation 1348 m) (Fig. 5) to assess the potential of biochar and compost amendments to improve soil health and mitigate climate change in semi-arid cropping systems. The study site has Olton clay loam soil (i.e., fine, mixed, super-active, thermic Aridic Paleustolls; Table S3), and a semi-arid climate^68^. The 72-year average annual precipitation and average annual temperature at the experimental site are 42.7 cm and 14.1 °C^69^. July and January had the highest (24.7 °C) and lowest (3.11 °C) average temperatures, and August and January experienced the highest and lowest precipitation of 7.70 and 0.91 cm, respectively, according to 25-year average data^70^.

The experiment was established in a randomized complete block design with four treatments and four replications. The treatments compared were the field application of pristine biochar, compost, a pristine biochar-compost mixture (BC), and an unamended control (CTRL). The individual plot size was 112 m^2^ (9.14 m x 12.2 m). The biochar was made from southern pine wood (*Pinus taeda* L.) pyrolyzed in commercial kiln at 60 °C as described previously[84]. It contained 788 g kg^− 1^ carbon, 2.67 g kg^− 1^ nitrogen (N), and 0.13 g kg^− 1^ sulfur. Volatile matter and ash contents were 119 g kg^− 1^ and 185 g kg^− 1^, respectively, and a specific surface area was 364 m^2^ g^− 1^ (Table S4 & S5). Compost was obtained from a local dairy in Clovis, NM, and contained 102 g kg^− 1^ soil organic matter, 0.93 g kg^− 1^ nitrate-N, 0.37 g kg^− 1^ available phosphorus, 10.4 g kg^− 1^ potassium, and 0.75 g kg^− 1^ sulfate-S. Biochar and compost were applied at a rate of 5 Mg ha^− 1^ on March 20, 2024, using a compost spreader. The BC treatment consisted of a combined application of 5 Mg ha^− 1^ biochar plus 5 Mg ha^− 1^ compost. The application rates were based on their effectiveness in improving soil properties and agronomic yield, as reported in previous studies^71^. No soil amendments were applied to the CTRL treatment. After soil application, the amendments were incorporated into the soil by shallow (5 cm) vertical tillage with no-till drill coulters. The plots were managed under no-tillage practices thereafter.

Sorghum [*Sorghum bicolor* (L.) Moench] (Variety P8620) was planted June 17, 2024. Fertilizer was applied at planting (112 kg ha^− 1^ N, 39 kg ha^− 1^ P, 18 kg ha^− 1^ S), with equal amounts applied in each experimental plot regardless of the treatments. Irrigation water (limited irrigation) was applied through a center pivot system at 2, 5, 30, 45, 58, 67, and 80 days after planting, representing various critical growth stages of the crop, with respective application amounts of 25.4, 25.4, 45.7, 38.1, 45.7, 45.7, 45.7, and 38.1 mm. These irrigation events cover the seedling emergence, growing point differentiation, boot stage, and soft dough stages of sorghum. Weeds were controlled by herbicides as needed. For example, a day before planting, a mixture of Atrazine [Atrazine, 42.6% a.i.] at the rate of 1.38 L ha^− 1^, Roundup Power Max [N-(phosphonomethyl glycine, 48.7% a.i.] at the rate of 2.22 L ha^− 1^, and Accuvart at the rate of 0.30 L ha^− 1^ was applied. On July 16, 2024, herbicide Warrant [acetochlor, 33% a.i] at the rate of 4.69 L ha^− 1^ was applied. Sorghum was harvested on October 16, 2024, using an International 1480 Combine harvester^®^. Grain yield was estimated by harvesting two center rows of 3.05 m length (4.65 m^2^ total area) in each plot. Crop production was measured by collecting aboveground biomass samples from a single 10-ft row in the plot center following grain harvest.

### Greenhouse gas measurements

**Fig. 6:**
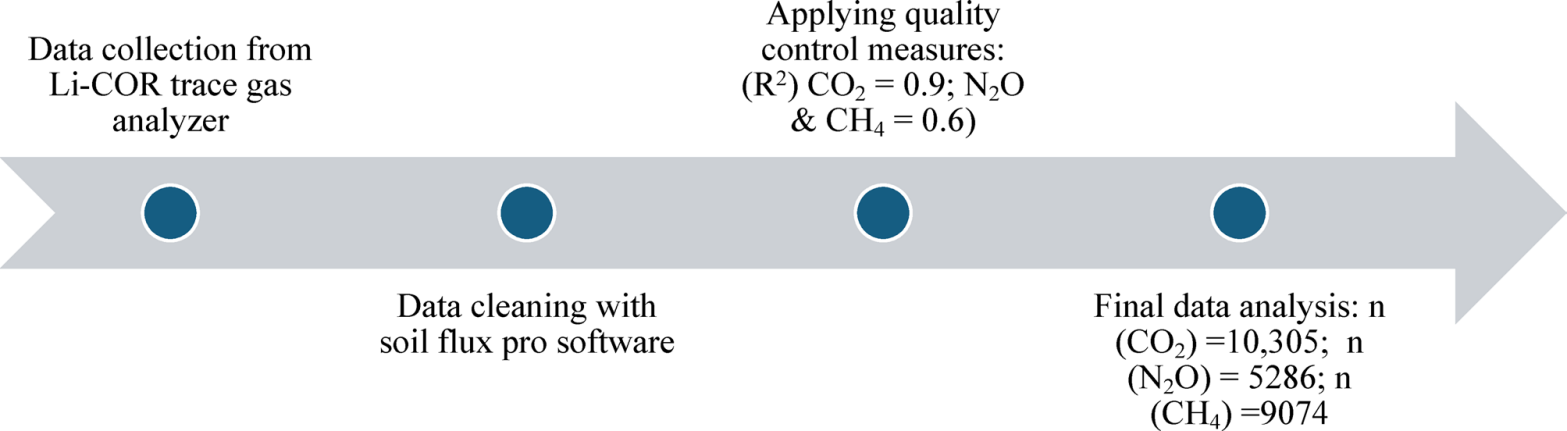
Greenhouse gas emission data processing pipeline. The total number of data points (n) for each gas differed after removing the odd data points.

Soil N_2_O, CO_2_, and CH_4_ emissions were measured every two hours throughout the study using automated LI-COR 8100–104 long-term chambers connected to LI-7810 CH_4_/CO_2_/H_2_O and LI-7820 N_2_O/H_2_O trace gas analyzers controlled by a LI-8250 Multiplexer (LI-COR Biosciences, Lincoln, NE, USA). The gas emissions were measured during April to October 2024 from two replications of pristine biochar and CTRL treatments and unreplicated compost and BC treatments. Each chamber was placed on a polyvinyl chloride (PVC) collar (inside diameter: 20.1 cm; height: 11.4 cm) and remained in place throughout the experimental period, except during occasional field operations (i.e., tillage and spraying). These chambers were installed between plant rows to minimize plant-derived GHG emissions entering the chambers during measurements. When chambers were removed for field operation, they were reinstalled immediately afterward. The chambers enclosed a constant volume of air, and GHG concentrations were measured over 2 min at every 2-hour interval. The collar height above the soil surface was maintained at 5 cm. A top-mounted pressure vent in the automated chamber prevents pressure spikes upon chamber closure, ensuring equilibrium with ambient atmospheric pressure under variable wind conditions. The gas analyzers connected to the long-term chambers measured the GHG emissions in nmol m^− 2^ s^− 1^ or µmol m^− 2^ s^− 1^ during the period of chamber closure (Eq. 1).

1 $$F = \frac{{10V~P~\left( {1 - \frac{W}{{1000}}} \right)}}{{RS\left( {T + 273.15} \right)}}~ \times \frac{{\partial C^{\prime } }}{{\partial t}}$$

where *F* is the soil GHG efflux rate (µmol m^− 2^ s^− 1^ ), *V* is volume (cm^3^), *P* is the atmospheric pressure (kPa), *W* is the water vapor mole fraction (mmol mol^− 1^), *S* is soil surface area (cm^2^), *T* is the air temperature (°C), and $\frac{\partial{C}^{{\prime}}}{\partial t}$ is the rate of change in water vapor dilution corrected GHG mole fraction (µmol mol^− 1^ s^− 1^). The analyzers use Optical Feedback-Cavity-Enhanced Absorption Spectroscopy to measure the gases collected by the chambers (LI-COR Biosciences, Lincoln, NE, USA). An in-built sensor in the chamber also measured air temperature. In addition, soil moisture sensors (i.e., Steven Hydra Probe) were connected to chambers to record soil water content and soil temperature at 5 cm depth concurrently with gas flux measurements. SoilFlux Pro software (LI-COR Biosciences Inc.) was used to process the data. Gas concentrations were adjusted to the linear fit criterion, and various coefficients of determination (*R*^2^) values were used for the data quality control (Fig. 6) based on the literature. For example, CO_2_ fluxes with *R*^2^ < 0.9 and N_2_O and CH_4_ fluxes with *R*^2^ < 0.6 were considered poor fits, indicative of unsuitable chamber conditions for gas measurements^72^. Further, observed data were divided into two seasons: no-crop season (April–June) and crop season (July–October) to assess the seasonal variability in GHG emission rates.

### Statistical analysis and net global warming potential calculation

The GHG data were statistically analyzed using a Generalized Additive Model (GAM) in R 4.5.0 (R Core Team, 2025, Vienna, Austria) via the mgcv package to assess temporal patterns in GHG emissions and environmental parameters. The GAM model allows for estimating nonlinear relationships between response and predictor variables through a smoothing function and can be used for non-replicated repeated-measures observations^73^. All fitted GAMs were created using cubic regression splines, which enable the modelling of non-linear data and reduce the risk of over-fitting. This model estimated the influence of soil water content, soil temperature, and treatments on GHG fluxes while accounting for repeated measures (Date) as random effects and treatments as fixed effects. The initial model was fitted with a Gaussian distribution, but the residuals indicated a non-normal distribution, with strongly right-skewed data and both positive and negative values for N_2_O–N, CO_2_–C, and CH_4_–C fluxes. Therefore, log-transformed data were refitted to the model to reduce the heteroscedasticity and non-normality. Model predictions and confidence intervals (95%) were estimated from GAM and plotted to identify any significant variation in GHG emissions with soil water content and soil temperature in each treatment. Other statistical summaries were computed using base R functions and the dplyr package. In this study, only descriptive statistics are presented because treatment replication was limited and unbalanced due to the high budgetary costs.

Cumulative emissions of all the gases were calculated through a linear interpolation of the bi-hourly data and numerical integration of individual data points. Net GWP for each treatment was estimated by quantifying the radiative forcing associated with soil GHG emissions (CO_2_, CH_4_, N_2_O) due to field management, soil amendment application, farm inputs, and soil-crop-microbe interactions. The CO_2_–C equivalent emission for each treatment was calculated based on a 100-year time scale using the conversion factors of 273 for N_2_O and 28.5 for CH_4_^29^. Net GWP $\left(kgC{O}_{2}eh{a}^{-1}yea{r}^{-1}\right)$ was calculated by using the soil heterotrophic respiration method, in which the CO_2_e emissions from both farm inputs and outputs were estimated (Eq. 2)^22,65^. Net GWP is calculated mostly through the Soil Organic C method (19–91% uncertainty), while the soil respiration method poses 27–95% uncertainty^65^. However, in our study soil respiration method is considered most appropriate, as detecting measurable changes in overall soil organic carbon content might require a period exceeding at least 2 years^65^.

2 $$NetGWP=(GW{P}_{inputs}+GW{P}_{N2O}\pm GW{P}_{CH4}+GW{P}_{RH}+GW{P}_{CP}-(GW{P}_{PYCR}+GW{P}_{CIamendment})$$

The *GWP*_*inputs*_ were estimated by summing the CO_2_e of farm operations (machinery operations for field activities), irrigation, compost, biochar, herbicide applications, input transportation, and aboveground biomass production, following the approach outlined by West and Marland^74^ and the irrigation and fertilization approach by Borsato et al.^75^. The CO_2_e for both center pivot operation and water pumping from on-farm pump, storage, and distribution were accounted for to quantify irrigation effects on GHG emissions^74^. Soil N_2_O emissions from fertilizer application were calculated considering both direct and indirect effects (N from chemical and organic fertilizers, and crop residue), as the amount of N_2_O emitted from ammonia volatilization and NO_3_^−^ leaching is 0.4% of the N fertilizer applied, with a 40–110% uncertainty^65^. Compost-related emissions were estimated for a 90-day storage period^76^. Net CO_2_e from biochar manufacturing was calculated according to the values described by Desjardins et al.^77^, where 1 Mg of biochar accounts for 0.0498 Mg of CO_2_e in the manufacturing process. The GWP N_2_O–N and CH_4_–C was estimated by quantifying cumulative N_2_O–N and CH_4_–C fluxes and estimating their CO_2_e emissions (Eq. 3).

3 $$CO_{2}Eqv. emissions=Cum. GHG\,Flux*Mol.wt. ratio*C{O}_{2}-CEqv.$$

Root respiration (RR) for the sorghum crop was assumed as 81.8% of total soil respiration for our calculations based on closed-chamber measurements^78^. Therefore, soil heterotrophic respiration (*RH*; kg CO_2_ ha^− 1^ year^− 1^) was calculated using Eq. 4, assuming the measured CO_2_ flux generated from both root and heterotrophic respiration, excluding plant respiration:

4 $$RH=\left(1-RR\right)*Cumulative\,C{O}_{2} Emission$$

where *RR* is the root respiration. The GWP from crop production (*GWP*_*cp.*_) was calculated based on the dry matter yield (crop residue and grain), assuming 0.34% of organic C content in the dry matter^79,80^. The GWP for previous years’ crop residues (GWPPYCR) was estimated by accounting for the decomposition of both below-ground and above-ground biomass, excluding grain removal. Total root-derived C was calculated by assuming root biomass of 60% of the total crop production^79^ as in Eq. 5:

5 $$Total\,root C=0.6\left\{\left[Grain\,yield*0.34\right]+\left[Aboveground\,Crop\,Residue*0.34\right]\right\}$$

It should be noted that the sorghum root: shoot C ratio is highly variable within sorghum varieties and agronomic conditions. Here, we assumed an average of 0.6, based on reported values from the literature^81,82^. Total root C accounts for root biomass produced by the plant and C from rhizodeposition. The previous year’s root biomass was calculated from 2024 CTRL plot yield data. The total root C value was converted to CO_2_e with the conversion factor of 3.66. Net GWP for each treatment was calculated for a 1 ha land area, incorporating replications. However, net GWP did not account for the plant’s CO_2_ exchange. Yield-scaled net GWP, or greenhouse gas intensity (GHGI), was also calculated using net GHG emissions and crop yield (Eq. 6).

6 $$Yield-scaled\,net\,GWP\left(GHGI\right)=\frac{NetGWP}{Crop\,biomass\,or\,grain\,yield}$$

For sorghum, the dry weight of grain was used as the yield for this calculation. For biochar and compost amended plots, GWP_C inputs_ (Eq. 2) were calculated using the initial C content of both biochar (788 g C kg^− 1^) and compost (59.2 g C kg^− 1^), and the decomposition rate of 0.35% for biochar^83^ and 21.6% for dairy compost^67^.

## Supplementary Information

Below is the link to the electronic supplementary material.

Supplementary Material 1

## Data Availability

All data supporting the findings of this study are available within the paper and its Supplementary Information.

## Declarations

### Statement for standard protocol

All methods were carried out in accordance with the relevant guidelines and regulations of the United States Department of Agriculture and New Mexico State University. The study was conducted following the standard GHG emissions and soil and plant sampling protocol.
