# Supplementary material for: Contrasting effects of biochar and compost on greenhouse gas emissions and the global warming potential of semi-arid cropping systems
Source: Sci Rep. 2026 Mar 6;16:12380. doi: 10.1038/s41598-026-42554-4 (PMC13084001; doi:10.1038/s41598-026-42554-4)
Supplement: Supplementary file 1 — Supplementary Material 1 [file 41598_2026_42554_MOESM1_ESM.docx]

# **Contrasting Effects of Biochar and Compost on Greenhouse Gas Emissions and the Global Warming Potential of Semi-Arid Cropping Systems**

Piumi Madhuwanthi^1^, Rajan Ghimire^1,2^, Sundar Sapkota^2^, Shannon Norris-Parish^3^, and April Ulery^1^

^1^Department of Plant and Environmental Sciences, New Mexico State University, Las Cruces, NM, USA

^2^Agricultural Science Center, New Mexico State University, Clovis, NM, USA

^3^Department of Agricultural and Extension Education, New Mexico State University, Las Cruces, NM, USA

Correspondence: [rghimire@nmsu.edu](mailto:rghimire@nmsu.edu); 2346 State Road 288, Clovis, NM 88101, USA

**Table S1**: Net GWP values calculated for farm-related activities in each treatment.

| **Activities** | **Net GWP (CO_2_e kg ha^-1^ yr^-1^)** | | | |
| --- | --- | --- | --- | --- |
|  | **Biochar** | **Compost** | **Control** | **Biochar + Compost** |
| Inputs **^a^** | 7,580 | 10,068 | 7,022 | 10,627 |
| Soil N_2_O emissions | 264 | 1,718 | 554 | 1,539 |
| Soil CH_4_ emissions | -0.04 | -0.03 | -0.04 | -0.03 |
| Soil respiration | 5,502 | 4,920 | 5,445 | 5,141 |
| Crop production **^b^** | 19,225 | 17,883 | 19,360 | 17,077 |
| Past year crop residue **^c^** | 21,809 | 21,809 | 22,614 | 22,346 |
| C inputs from substrates **^d^** | 3,926 | 850 | 0 | 4,776 |
| Net GWP (Mg CO_2_e ha^-1^ yr^-1^) | 6.84 | 11.9 | 9.80 | 7.26 |
| GHGI kg CO_2_e per 1 kg of dry grain yield | 0.51 | 0.97 | 0.80 | 0.53 |

CO_2_e from inputs was calculated based on the following assumptions, as suggested in existing work. Energy and CO_2_ emissions associated with machinery operations were calculated as described by West & Marland ^69^, considering tillage (minimum tillage), planting, single cultivation, biochar application, compost application, pesticide application, harvesting, and transportation. Here, we assumed that the energy cost for biochar and compost applications is similar. Irrigation was supplied through a center pivot irrigation system. Energy and CO_2_ emissions associated with one-time irrigation were calculated by considering pumping, storage, and distribution from on-farm pumps 69, as well as the application through the center pivot ^70^. Throughout the study period, eight irrigation events occurred. GHG emissions due to the applied inputs were calculated considering fertilizers (N, P_2_O_5_, and Sulfur) and pesticides. Here, both direct and indirect effects (Ammonia volatilization and N leaching) of N fertilization are calculated as described by Sainju ^59^. The pesticide effect was based on West & Marland ^69^. CO_2_e for manufacturing inputs was calculated considering biochar manufacturing (feedstock collection to production) as described by Desjardins et al ^72^, compost storage period (three months assumed; ^71^), and pesticide production. Approximately 1.70 kg ha^-1^ of herbicide was applied, and 18.2 to 26.6 kg of CO_2_e per kg is produced (Pesticide Action & Agroecology Network, 2023).

**^b^** CO_2_e from crop production was calculated considering the amount of grain yield, following the method described by Sainju ^59^.

**^c^** Past year crop residue root C content was calculated considering the belowground biomass from the previous year (2023) and the aboveground crop residues added to the soil in 2024 (except for grains).

**^d^** CO_2_e from C inputs to soil was calculated considering the C content added to soil in the form of compost and biochar substrates and retained in soil after one year of decomposition.

**Table S2**: Summary CO_2_e for the no-crop season and crop season for each treatment from GHG emissions

| **Treatment** | **No-crop Season**  **CO_2_e (100-year scale) (kg ha^-1^)** | | | | **Crop Season**  **CO_2_e (100-year scale) (kg ha^-1^)** | | | | |
| --- | --- | --- | --- | --- | --- | --- | --- | --- | --- |
|  | **CO_2_** | **N_2_O** | **CH_4_** | **Sum of all gases** | **CO_2_** | **N_2_O** | **CH_4_** | | **Sum of all gases** |
| Biochar | 3,106 | 37.1 | -4.75E-3 | 3,143 | 14,723 | 118 | -20.2E-3 | 14,841 | |
| Compost | 3,830 | 548 | -3.50E-3 | 4,378 | 12,114 | 456 | -12.8E-3 | 12,570 | |
| CTRL | 2,076 | 98.0 | -6.21E-3 | 2,174 | 15,570 | 226 | -14.7E-3 | 15,796 | |
| BC | 3,371 | 230 | -2.17E-3 | 3,601 | 13,289 | 668 | -14.9E-3 | 13,957 | |

**Table S3:** Inductively Coupled Plasma Optical Emission Spectroscopy (ICP-OES) of pristine biochar

| **ICP-OES Components**  **(mg kg^-1^)** | **Pristine Biochar** |
| --- | --- |
| Ca | 13,032 ± 1195 |
| Cd | <0.01 |
| Cr | <0.01 |
| Cu | 19.4 ± 0.14 |
| Fe | 1,893 ± 249 |
| K | 2,313 ± 157 |
| Mg | 2,592 ± 201 |
| Mn | 544 ± 35.3 |
| Mo | <0.01 |
| Na | 292 ± 36.0 |
| Ni | <0.01 |
| P | 525 ± 87.6 |
| Pb | <0.01 |
| Zn | 38.9 ± 0.29 |

**Table S4:** Ultimate and proximate analysis of pristine biochar

| Sample | C % | | H% | | N% | S% | | Ash% | O%* | | O/C | | H/C | (O+N)/C | | C/N |
| --- | --- | --- | --- | --- | --- | --- | --- | --- | --- | --- | --- | --- | --- | --- | --- | --- |
| Pristine Biochar | 78.8 ± 1.94 | | 1.49 ± 0.16 | | 0.27 ± 0.03 | 0.01 ± 0.01 | | 18.5 ± 1.28 | 0.93 | | 0.01 | | 0.02 | 0.02 | | 296 |
| ** Calculated by difference* | | | | | | | | | | | | | | | | |
| Sample | | Moisture % | | Volatiles % ^d^ | | | Ash % ^d^ | | | Fixed Carbon % ^*^ | | BET Surface area (m^2^/g) ^d^ | | | BJH Pore volume (cm^3^/g) ^d^ | |
| Pristine Biochar | | 31.1 ± 0.50 | | 11.9 ± 0.72 | | | 18.5 ± 1.28 | | | 69.6 | | 364 | | | 0.02 | |
| ** Calculated by difference, ^d^ Calculated on a dry basis* | | | | | | | | | | | | | | | | |


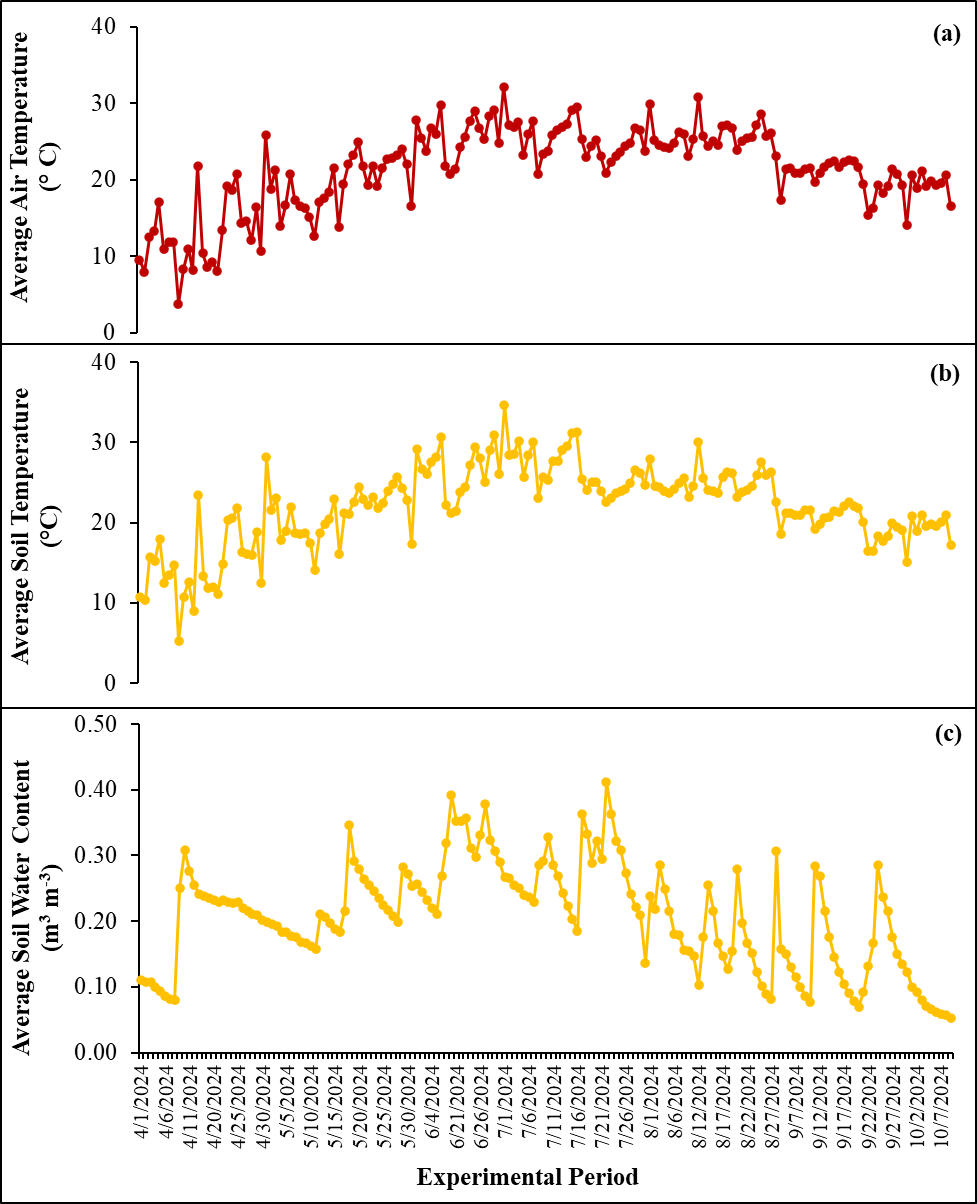


**Figure S1**: Average (a) air temperature, (b) soil temperature, and (c) soil water content throughout the experimental period (April – October 2024) across the treatments.


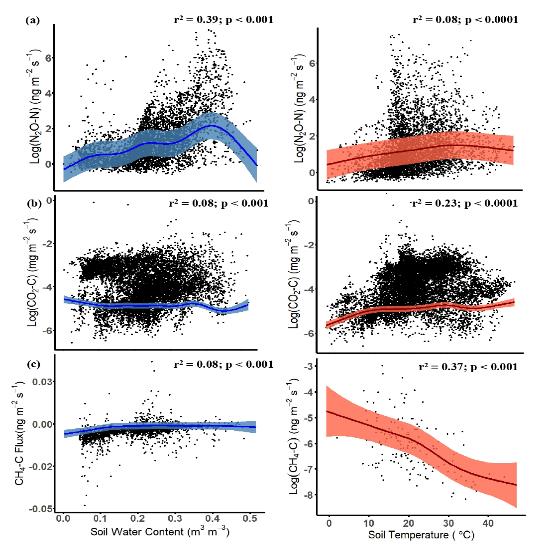


**Figure S2**: Nonlinear relationships of soil water content (blue line) and soil temperature (red line) against logarithmically transformed fluxes for (a) N_2_O-N, (b) CO_2_-C, and (c) CH_4_-C as explained by the GAM. Soil water content for CH_4_-C was plotted against non-log-transformed data. Scatter plots of individual observations across the full range of soil water content and soil temperature, overlaid with a smooth, fitted GAM curve generated using a cubic spline basis. The shaded area around the fitted line represents the 95% confidence interval at the 0.05 significance level.
